# Case Report of an Obstructive Hydrocephalus Caused by an Unruptured Mesencephalic Arteriovenous Malformation in a Boy and a Review of Literature

**DOI:** 10.2174/1874440001812010010

**Published:** 2018-02-21

**Authors:** Furkan Diren, Serra Sencer, Tayfun Hakan

**Affiliations:** 1 International Kolon Hospital, Neurosurgery Clinic, İstanbul, Turkey; 2 İstanbul University, İstanbul Medical School, Neuroradiology Department, İstanbul, Turkey; 3 Okan University, Vocational School of Health Services, İstanbul, Turkey

**Keywords:** Arteriovenous malformation, Aqueduct, Digital subtraction angiography, Hydrocephalus, Mesencephalon, Ventriculoperitoneal shunt

## Abstract

**Objective:**

Arteriovenous malformation (AVM) is the most common form of intracranial vascular malformations in adults. Intracranial pediatric AVMs are rare. AVM located in the vicinity of the brain stem in children are even more rare.

**Case report:**

This study reports a rare case of acute obstructive hydrocephalus following aqueductal stenosis caused by an unruptured grade IV perimesencephalic arteriovenous malformation. An 11-year-old boy admitted to the hospital with progressive headache, nausea and vomiting throughout a month. A Computerized Tomography (CT) showed an obstructive hydrocephaly. A Magnetic Resonance (MR) imaging revealed a mesencephalic AVM compressing the aqueduct. The patient deteriorated in hours and an emergency ventriculoperitoneal shunting was performed. He did well in the early postoperative period. AVM examined with Digital Subtraction Angiography (DSA) in detail for maintaining the definitive treatment by means of endovascular embolization, microsurgery and stereotactic radiosurgery; but the patient was lost to follow up.

**Conclusion:**

A Pubmed search revealed 34 cases of hydrocephalus caused by an unruptured AVM in the literature, and only four cases were less than 18 years old with unruptured AVM locating in brain stem or posterior fossa. Although focal neurologic deficit, seizure and headache are the most common symptoms, acute neurologic deterioration due to hydrocephalus may be the presenting symptom in these cases. The decrease in intracranial pressure by changing the flow of cerebrospinal fluid (CSF) via an emergency ventriculoperitoneal (VP) shunting or Endoscopic Third Ventriculostomy (ETV) can be a lifesaving procedure that gives a chance for further treatment modalities.

## 1 INTRODUCTION

AVM is the most common form of intracranial vascular malformations in adults. Intracranial pediatric AVMs are rare [1, 2]. AVM located in the vicinity of the brain stem in children are even more rare [1, 3]. Hemorrhage is the most common presentation of intracranial AVMs in children [3]. Focal neurological deficit, seizure and headache are the most common symptoms for unruptured AVMs in children [4]. An unruptured AVM presenting with an acutely worsening hydrocephalus is a rare event. We found only 34 cases of hydrocephalus caused by an unruptured AVM in the literature, and only eight of them were less than 18 years old and only 12 cases were locating in midbrain or posterior fossa (Table **1**).

This study illustrates a case of an unruptured pediatric perimesencephalic AVM that presented with acute neurological deterioration due to acute obstructive hydrocephalus in a child.

## 2 CASE REPORT

**Fig. (1):**
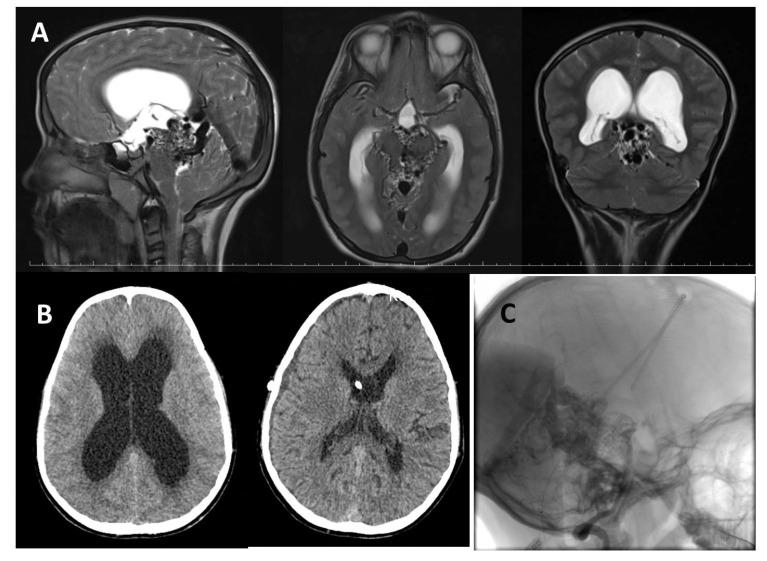
**A**) T2 weighted MR scans showing a mesencephalic AVM that caused hydrocephalus with obstruction of aqueduct in sagittal, axial and coronary planes. **B**) A control CT showing the enlarged lateral ventricle with transependymal edema (left), and the collapsed ventricles following VP shunt procedure, with tip of the ventricular catheter (right). **C**) A DSA view perimesencephalic AVM in lateral plane, with intraventricular catheter.

An 11-year-old male from abroad admitted to outpatient clinic with progressive headache, nausea and vomiting which had been presented for a month. Neurologic examinations showed bilateral papillary edema and right-sided central facial nerve palsy. He had been investigated for right sided facial palsy at home and was diagnosed with an AVM located in the perimesencephalic region two years ago. Head CT showed enlargement of lateral and third ventricles with transependymal cerebrospinal fluid oozing (Fig. **1B**). A prompt cranial MR revealed serpiginous signal void structures around the midbrain compatible with an AVM and enlargement of the vein of Galen and internal cerebral veins causing mechanical compression of the aqueduct causing an acute obstructive hydrocephalus caused (Fig. **1A**). Cerebral DSA was planned for diagnosis and treatment planning, however; due to rapid deterioration in patient’s consciousness DSA was postponed and emergency right VP shunting was done in the same day. His conciseness improved, headache and vomiting disappeared immediately in the early postoperative period. Control CT scan showed good ventricular decompression (Fig. **1B**). DSA was performed *via* right femoral access two weeks after the operation. Bilateral injection of internal carotid arteries showed enlarged posterior cerebral arteries bilaterally, supplying a diffuse AVM by the perforating arteries of the posterior lateral, medial choroidal and posterior communicating arteries. Venous drainage was into enlarged internal cerebral veins and vein of Galen. There was also some reflux to the deep tentorial and adjacent cortical veins due to high flow. The late capillary and venous phase was prolonged because of venous hypertension (Fig. **1C**). The AVM was assessed as grade IV according to the Spetzler-Martin AVM grading system. A multimodal treatment protocol including endovascular embolization and stereotactic radiosurgery to portions of residual AVM was suggested. Patient was discharged on his family’s request as neurologically intact except for a mild right-sided facial palsy and was lost to follow-up.

## 3 DISCUSSION

### 3.1 Demographic Features

Hydrocephalus caused by an unruptured AVM may be seen in both gender and in every age but rare less than 18 years [5-8]. Only 4 cases of pediatric patients -two female & two male- with hydrocephalus and unruptured AVM locating in midbrain or posterior fossa were reported in the literature [5, 8, 9]. We added the fifth pediatric patient with this study.

### 3.2 Clinical Signs and Symptoms

Hydrocephalus is mostly expected to be happening after the hemorrhage of an AVM into the ventricular system or subarachnoid space [10]. Hydrocephalus is an uncommon neurologic problem in patients with unruptured AVMs [5]. Presentation of a deeply located intracranial unruptured AVM with acute obstructive hydrocephalus is a rare entity in children. Hemorrhage is usually seen with deep seated AVMs due to deep venous drainage and in unruptured AVMs mostly located in cerebral hemispheres in children [4]. In this case however, the AVM was located around the midbrain and not caused hemorrhage. Hemorrhage, seizure, headache and focal neurologic deficits are common neurologic problems at presentation in children with intracranial AVMs [11, 12]. As hemorrhage is the most common symptom in ruptured AVMs, focal neurologic deficit, seizure and headache commonly seen in unruptured AVMs [4, 13]. Incidental AVMs may also be encountered during childhood deterioration [11]. Focal neurologic deficit can be seen in patients with ruptured or unruptured AVMs, but acute neurologic deterioration due to hydrocephalus with unruptured AVM is a rare entity [7, 10]. The presented case had a cranial nerve deficit for nearly two years, but acute neurological deterioration due to hydrocephalus ensued over a short period of hours. According to the patient’s history of the presented case, an intracranial AVM was detected following facial palsy two years ago, and no new sign or symptom added until one month ago. Although the mechanism -like enlargement as a hemodynamic consequence of increased flow, or stimulated proliferation because of shunt- has been a matter of controversy, the increase in size of AVM has been documented [14]. We do not have any knowledge about the size of previously found AVM, but we can suggest that the occurrence of obstructive hydrocephalus and deterioration of the patient is most probably due to enlargement of the size of mesencephalic AVM.

### 3.3 Cause of Hydrocephalus

Hydrocephalus can occur as a result of the venous outflow and hemodynamic unbalance [5, 8, 10]; or mechanical obstruction of the ventricles by drainage vein or AVM [5, 7], or by compression of the aqueduct [5, 15-17] in unruptured AVM cases. Ebuni *et al.* [18] suggested that hydrocephalus was result of reflux into periventricular and transmedullary veins instead of mechanical obstruction in their study. Overproduction of cerebrospinal fluid may also be a cause of hydrocephalus in an unruptured choroidal AVM case [19]. In the presented case, the hydrocephalus was thought to be due to mechanical obstruction of the aqueduct by AVM.

### 3.4 Management of Obstructive Hydrocephalus Due to an AVM

Microsurgical resection [11], endovascular embolization [5, 12], stereotactic radiosurgery [4, 5], and/or multimodal therapy [1] consist of current intracranial AVM treatment. Treatment of brainstem AVMs -especially with high (Grade IV and V) Spetzler-Martin grade, is a challenge [9, 20]. Removal of brain stem AVMs with microsurgical resection is difficult and entangled with a high surgical risk. Since they are located deeply and close to vital structures, radiosurgery also has high risk of adverse effect and hemorrhage during latency periods, and rate of obliteration is relatively low. Preoperative embolization is advised before microsurgical excision for grad IV-V unruptured AVMs locating in brainstem [20]. In case of AVM with large size or locating in eloquent area, conservative treatment may be adopted [5].

Obstructive hydrocephalus due to an AVM can be treated by a VP shunting [5, 18, 21] or ETV [6, 15, 22]. ETV is a technique which would not involve a change between supratentorial and infratentorial pressure relationships [7] and it considered as an advantage for being “shunt free” [15]. Malfunctioning or over drainage may be seen following VP shunting [5]. We chose VP shunting in this case because that procedure was the fastest treatment option in the emergency setting in our conditions [5] to treat obstructive hydrocephalus.

Some authors primarily chose to treat AVM in cases with mild symptoms of obstruction that not required emergency [18, 23].

Patient’s family requested discharge although they were fully informed about the disease and treatment options due to financial concerns and patient was lost to follow up.

## CONCLUSION

Presentation of a deeply located intracranial unruptured AVM with acute obstructive hydrocephalus is a rare entity in children. Although focal neurologic deficit, seizure and headache are the most common symptoms, acute neurologic deterioration due to hydrocephalus may be the presenting symptom in these cases. The decrease in intracranial pressure by changing the flow of CSF *via* an emergency VP shunting or ETV can be a lifesaving procedure that gives a chance for further treatment modalities.

## Figures and Tables

**Table 1:** Literature Review of Hydrocephalus due to Unruptured AVM.

**Author**	**Age & Sex**	**Localization of AVM**	**Treatment of hydrocephalus**
Geibprasert *et al.* [5]	16, F	Thalamus	VP shunting
	17, F	Bazal ganglia & thalamus	VP shunting
	35, M	Temporoparietal	VP shunting
	39, F	Frontal /callosum	None
	6, F	Midbrain	VP shunting
	26, M	Cerebellum	VP shunting
	55, M	Cerebellum	VP shunting
	2, M	Cerebellar vermis	None
Park *et al.* [6]	0, M	Cerebraal hemispheres	None
Rodrigez and Molet [7]	83, M	Posterior fossa	ETV
	64, F	Posterior fossa	ETV
Montoya *et al.* [8]	0, F	Torcula	Ventriculojugular shunting
	5, F	Thalamus	VP shunting
Nozaki *et al.* [9]	0, M	Midbrain	VP shunting
Mindea *et al.* [10]	55,M	Parietoocciptal and Galenic region	VP shunting
Millar *et al.* [13]	?, M	NA	NA
	?, M	NA	NA
	?, M	NA	NA
	?, M	NA	NA
	?, F	NA	NA
Champeaux *et al.* [15]	54, M	Thalamic insular & capsular	ETV
Ono *et al.* [16]	56, M	Cerebellar vermis	ETV
Tucker *et al.* [17]	63, M	Pineal region	ETV
Ebinu *et al.* [18]	61, M	Periventricular	None
Bayri *et al.* [21]	37, M	Frontal	VP shunting
Rezaee *et al.* [22]	NK	NK	ETV
Pribil *et al.* [23]	20, M	Fronto-parietal	None
DeFoe *et al.* [24]	24, M	Mesencephalon	VP shunting
Esparza *et al.* [25]	20, F	Posterior fossa	None
Hoi & Kerber [26]	31, F	Thalamus	VP shunting
Kurita [27]	NA	NA	NA
Liu *et al.* [28]	49, M	Thalamus	Endovascular intervention
Lobato *et al.* [29]	42, F	Mesencephalon	VP shunting
Pereira *et al.* [30]	41, M	Pineal region	ETV
Present case	11, M	Perimesencephalic	VP shunting

ETV: Endoscopic Third Ventriculostomy, NA: Not Available, NK: Not Known, VP: Ventriculoperitoneal
